# Cardiac Amyloidosis Treatment

**DOI:** 10.14797/mdcvj.1050

**Published:** 2022-03-14

**Authors:** Lily K. Stern, Jignesh Patel

**Affiliations:** 1 Smidt Heart Institute, Cedars-Sinai, Los Angeles, California, US

**Keywords:** cardiac amyloidosis, transthyretin amyloidosis, light chain amyloidosis, monoclonal light chains, amyloidosis treatment, autologous stem cell transplantation, daratumumab, tafamidis, patisiran, inotersen

## Abstract

Cardiac amyloidosis (CA) is a restrictive cardiomyopathy with a traditionally poor prognosis. Until recently, CA treatment options were limited and consisted predominantly of managing symptoms and disease-related complications. However, the last decade has seen significant advances in disease-modifying therapies, increased awareness of CA, and improved diagnostic methods resulting in earlier diagnoses. In this review, we provide an overview of current and experimental treatments for the predominant types of CA: transthyretin cardiac amyloidosis (ATTR-CA) and immunoglobulin light chain (AL)-mediated CA (AL-CA).

The mainstay of AL-CA treatment is proteasome inhibitor-based chemotherapy with daratumumab and, when feasible, autologous stem cell transplantation. For ATTR-CA, the stabilizer tafamidis is the only US Food and Drug Administration (FDA)-approved treatment. However, promising novel therapies on the horizon target various points in the ATTR-CA amyloidogenic cascade. These include transthyretin gene (*TTR)* silencing agents to prevent TTR formation, TTR tetramer stabilization and inhibition of oligomer aggregation to prevent fibril formation, anti-TTR fiber antibodies, and amyloid degradation. For end-stage CA, advanced interventions may need to be considered, including heart, heart-kidney, and, for hereditary ATTR-CA, heart-liver transplantation. Despite the evolution of treatment options, CA management remains complex due to patient frailty and therapeutic side effects or intolerance with advanced cardiac disease. This is particularly relevant for those with AL-CA, when active teamwork between the hematologist-oncologist and the cardiologist is critical for treatment success. Often, referral to an expert center is necessary for timely diagnosis, initiation of treatment, and participation in clinical trials.

## Introduction

**Figure 1:**
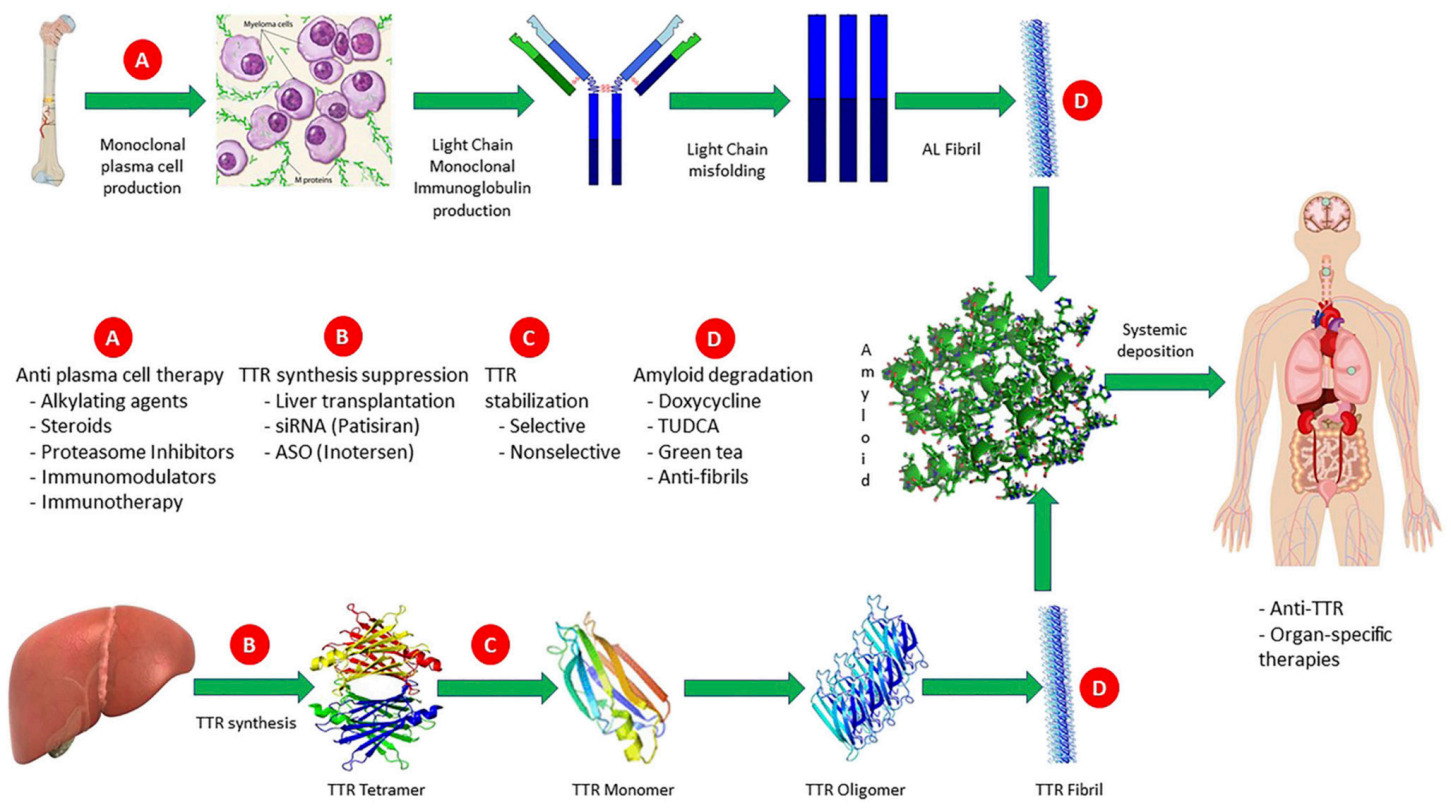
Targets of treatment along the light chain and transthyretin amyloidogenic pathway. Reproduced with permission from @John Wiley & Sons Ltd on behalf of European Society of Cardiology, Adam et al.^1^ AL: amyloid light chain; TTR: transthyretin; siRNA: small interfering ribonucleic acid; ASO: antisense oligonucleotide; TUDCA: tauroursodeoxycholic acid

Cardiac amyloidosis (CA) is a rare and progressive disease resulting from protein buildup in cardiac muscle. Treatment options for CA were previously limited to symptom management. Over the last decade, however, there have been significant advances in disease-modifying therapies that provide hope for slowing disease progression to optimize quality of life and improve survival. Here we provide an overview of novel and experimental treatment strategies for the predominant types of CA, transthyretin cardiac amyloidosis (ATTR-CA) and immunoglobulin light chain (AL)-mediated CA, or AL-CA (***Figure 1, Table 1***).^1,2^

**Table 1:** General treatment strategies for cardiac amyloidosis subtypes. Adapted from @Springer Science + Business Media LLC, Stern and Kittleson.^2^ GDMT: guideline-directed medical treatment *with ace-inhibitors, angiotensin receptor blockers, angiotensin receptor blocker-neprilysin inhibitor, beta-blockers, aldosterone antagonists, sodium glucose cotransporter-2 inhibitors; AF: atrial fibrillation or atrial flutter; DOAC: direct oral anticoagulant; VKA: vitamin-K antagonist; PPM: permanent pacemaker; ICD: implantable cardioverter defibrillator; VT: ventricular tachycardia; SCD: aborted sudden cardiac death; HRS: Heart Rhythm Society; AL: light chain amyloidosis; ATTRv: hereditary transthyretin amyloidosis; ATTRwt: wild-type ATTR amyloidosis; CM: cardiomyopathy; PN: polyneuropathy; PO: per oral administration; SQ: subcutaneous administration; IV: intravenous administration; FDA: Food and Drug Administration; CyBorD: cyclophosphamide-bortezomib-dexamethasone; BMD: bortezomib-melphalan-dexamethasone; ASCT: autologous stem cell transplant


TREATMENT CATEGORY	TREATMENT	COMMENTS AND CAVEATS

Heart failure	Loop diuretics	Favor bioavailable (bumetanide, torsemide)

	GDMT* if tolerated	Clinical benefit not established May be poorly tolerated due to restrictive physiology and renal dysfunction

Autonomic dysfunction	(1) Midodrine (2) Droxidopa (3) Pyridostigmine (4) Compression stockings	(1–3) Usually AL-CA and ATTRv-CA (3) Not formally studied in CA (4) For orthostasis and mobilization of peripheral edema for all types of CA

**Arrhythmias**		

Medical	Amiodarone (AF)	Usually tolerated over nodal blocking agents due to tendency for conduction disease and heart rate dependence; no difference for rate or rhythm control

	Anticoagulation (AF)	DOAC or VKA Prescribed regardless of CHA2DS2-VASc score

Device	PPM (Heart block)	CRT may be considered in select PPM-dependent patients

	ICD (VT/SCD)	Heart Rhythm Society recommendation^75^: Primary prevention: AL-CA with NSVT with > 1 yr life expectancy (IIb) Secondary: > 1 yr life expectancy (Ic)

Advanced therapies	Heart transplant	AL-CM: select patients with good response to chemotherapy/immunotherapy and minimal extracardiac involvement ATTR-CM: Select patients with minimal extracardiac symptoms

	Heart-liver transplant	ATTRv-CM + PN: liver transplant may be unnecessary in the future with advances in silencer therapy

**Currently available disease-modifying therapy**		

**ATTR-CA**		

ATTRwt-CM	(1) Tafamidis (2) Diflunisal	TTR stabilizers: halts disease progression PO tablets (1) FDA approved (2) Off-label, NSAID: contraindicated for renal failure and thrombocytopenia; used cautiously with anticoagulation and gastrointestinal bleed

ATTRv-CM	(1) Tafamidis (2) Diflunisal	

ATTRv-CM + PN	(1) Tafamidis (2) Inotersen (3) Patisiran (4) Diflunisal	TTR stabilizers: (1) FDA approved (4) off-label TTR silencers: prevent amyloid formation (2) SQ, risk of thrombocytopenia and glomerulonephritis (3) IV, fewer reported side effects

ATTRv-PN	(1) Inotersen (2) Patisiran (3) Diflunisal	TTR silencers (1,2) TTR stabilizer (3) off label

**AL-CA**		

Chemotherapy	CyBorD/BMD	Most common chemotherapy regimens in patients who are not candidates for ASCT and as up-front therapy with daratumumab Corticosteroids/volume of therapy may precipitate decompensated heart failure

Immunotherapy	Daratumumab	Anti-CD38 monoclonal antibody Only FDA-approved therapy for AL amyloidosis Up-front therapy combined with bortezomib-based chemotherapy regimens

	(1) Lenalidomide (2) Pomalidomide (3) Thalidomide	Thalidomide analogs (1–3): Generally reserved for relapsed disease Potential cardiac and renal toxicity

Transplant	High-dose melphalan + ASCT	Preferred approach but rarely offered if significant cardiac and/or other organ involvement May be performed after heart transplantation


## Disease-Modifying Treatment for AL Cardiac Amyloidosis

AL-CA results from misfolded immunoglobulin light-chain proteins that infiltrate cardiac muscle and lead to organ failure. Since extent of light chain fibril deposition and resultant toxicity drives AL-CA disease severity and prognosis, the aim of treatment is to eradicate the underlying plasma cell clone that produces the excess light chains. Chemotherapy regimens and autologous stem cell transplantation (ASCT) have been adapted from treatment of multiple myeloma, a related plasma cell dyscrasia. While treatment is directed by the hematologist-oncologist, many regimens have significant toxicities that are poorly tolerated in patients with cardiac involvement, so it is critical to have a close alignment with the cardiologist on the treatment team.

The goal of treatment is to administer the strongest chemotherapy regimen that the patient can safely tolerate with the intent of improving organ function and survival.^3,4^ Even in advanced cardiac disease (Mayo cardiac stage IIIb), early response at 1 month has been associated with improved overall survival.^5^ Success is determined by hematologic response (suppression of serum free light chains) and organ-specific response (evaluation of cardiac and renal biomarkers). A recently validated composite hematologic/organ response model^6^ classifies patients into two treatment response groups to determine early clinical benefit, and it has shown a higher prognostic power than assessment of isolated hematologic or organ response. Rapid improvement in left ventricular (LV) thickness and function is uncommon after treatment regardless of improvement in cardiac biomarkers. Global longitudinal strain and cardiac magnetic resonance imaging with T1 mapping and extracellular volume measurements provide more subtle evidence of structural treatment response.^7,8^

### Up-Front Amyloidosis Therapy

Risk stratification dictates initial treatment strategy for AL-CA patients. High-dose melphalan with ASCT provides the greatest opportunity for long-term remission. However, to qualify for ASCT as initial therapy, patients must be low risk, defined as age < 70 years old with good functional status (Eastern Cooperative Oncology Group performance status [ECOG PS] < 2) and without significant organ dysfunction, including New York Heart Association (NYHA) class < III and biomarker levels below validated thresholds. Due to disease severity and comorbidities at the time of diagnosis, less than one in five patients are eligible for up-front ASCT. If there is high bone marrow plasma cell infiltration (> 10%) and/or any anticipated delay in initiation of ASCT, patients may require induction therapy with the proteosome inhibitor bortezomib, with dexamethasone and an alkylating agent. Bortezomib-based induction therapy has been shown to improve progression-free survival with an 84% hematologic response (39% complete response) and an 84% overall 5-year survival.^9^ Bortezomib-based therapies are also used after ASCT for consolidation treatment if there is less than a very good partial response.

For intermediate risk (cardiac stage I-IIIa)^10^ patients who initially do not qualify for ASCT, chemotherapy is initiated, and ASCT can be reconsidered if reversible contraindications improve. For many years, the standard chemotherapy regimen was high-dose melphalan with dexamethasone, but the addition of bortezomib in recent years has become the standard of care, with significantly improved hematologic response and overall survival in a phase 3 randomized open-label trial.^11^ The two most common regimens are cyclophosphamide-bortezomib-dexamethasone (CyBorD) and bortezomib-melphalan-dexamethasone. Recently, the anti-CD38 human IgG-κ monoclonal antibody, daratumumab, previously used for relapsed or refractory AL, has also become part of the standard up-front regimen. The ANDROMEDA phase 3 trial showed that subcutaneously administered daratumumab plus CyBorD compared with CyBorD alone had more rapid and significant hematologic response (53% vs 18%) and improved survival from major organ deterioration or hematologic progression (HR 0.58; CI 0.36-0.93) for newly diagnosed AL.^12^ Although there was an overall increase in lymphopenia and severe infections, the incidence of overall severe adverse events was lower in the daratumumab group when adjusted for exposure to trial treatment. Of note, most of the trial population had NYHA class II (40%) and NYHA Class IIIa (34%) symptoms. In a prespecified subgroup analysis, daratumumab demonstrated an escalating hematologic response with higher NYHA class, suggesting particular efficacy and safety for those with higher-grade cardiac involvement. After the ANDROMEDA trial, daratumumab with hyaluronidase-fihj became the first FDA-approved treatment for AL amyloidosis.^13^

High-risk patients with poor functional capacity (ECOG PS 4) and advanced cardiac involvement (NYHA class IIIb or IV) often do not tolerate standard chemotherapy and may require reduced doses or a sequential introduction of chemotherapy agents with frequent reassessment for potential aggressive escalation. Given the increasing evidence for daratumumab in this population, monotherapy daratumumab is being investigated for newly diagnosed stage IIIb AL in a phase 2 open-label trial.^14^

Contraindications to bortezomib include pulmonary fibrosis and peripheral neuropathy. Carfilzomib is another second-generation proteasome inhibitor that is beneficial in place of bortezomib for those with significant neuropathy; however, it is associated with a rise in NT-proBNP and cardiotoxicity and therefore not used for those with AL-CA.^15^ Additional consideration for regimen selection is the presence of genetic abnormalities. The chromosomal translocation 11; 14 is present in up to 60% of AL cases and is associated with poorer outcomes with bortezomib and immunomodulatory therapy (IMiD), whereas the gain of function 1(q21) has been associated with poorer response to melphalan. Alternative therapies for patients with contraindications to bortezomib are under investigation, including the oral selective B-cell lymphoma 2 inhibitor, venetoclax, for patients with t(11;14), although limited data are available.^16^

## Refractory or Relapsed AL Therapy

Patients who relapse or are refractory to up-front therapy are usually treated with daratumumab, if not already initiated, based on many promising retrospective and phase 2 trials.^17,18^ Otherwise, IMiD and low-dose dexamethasone with or without cyclophosphamide are used. The IMiDs lenalidomide, pomalidomide, and thalidomide have shown efficacy in retrospective and phase 2 clinical trials but have not been compared to other regimens.^19^ Lenalidomide and thalidomide treatment have been associated with thrombotic complications requiring thromboprophylaxis. IMiDs have been associated with a discordant rise in NT-proBNP levels despite improvement in free light chains, which poses challenges for cardiac response assessment.^20^ An all-oral regimen for relapsed or refractory AL with the proteasome inhibitor ixazomib plus dexamethasone was studied in the TOURMALINE-AL1 phase 3 study; while not associated with improved overall hematologic response, it demonstrated a higher complete response rate (26% vs 18%) and longer progression-free survival compared with physicians’ choice (47% lenalidomide plus dexamethasone).^21^ A non-IMiD–based rescue regimen with the alkylating agent bendamustine and dexamethasone has also been investigated in a phase 2 study and demonstrated a 57% hematologic response rate.

Immunotherapy with monoclonal antibody NEOD001 has also been investigated. However, the PRONTO phase 2b trial for AL-CA with refractory cardiac dysfunction failed to meet primary and secondary end points, and the VITAL phase 3 trial for treatment-naive AL-CA was discontinued based on futility analysis.^22^ Another chimeric monoclonal antibody, CAEL-101, which targets AL amyloid deposits, was found to be well tolerated in a phase 2 study^23^ and is now under investigation in two phase 3 trials in Mayo Stage IIIa^24^ and Stage IIIb^24,25^ disease.

### Adjunct AL Therapy

Epigallocatechin-3-gallate (EGCG), the most abundant catechin in green tea, is a major antioxidant that has been shown to stabilize misfolded amyloid fibrils into a less toxic form and prevent formation of insoluble amyloid fibrils.^26^ EGCG has been investigated in three phase 2 trials for treatment of AL-CA. Two of these trials have completed enrollment—EpiCardiAL^27^ and TAME-AL—but results had not been published at time of publication.^28^ A small single-center Japanese study of 57 patients did not report a significant benefit for use of EGCG but also did not report any significant toxicity.^29^ Because EGCG is well tolerated, it is often added as an adjunct to AL-CA treatment regimens.

The antibiotic doxycycline has been shown to disrupt AL amyloid fibril formation in transgenic mice and to counteract AL amyloid toxicity in C elegans.^30,31^ It showed promise in retrospective studies to improve hematologic response and overall survival.^32,33^ However, a recent prospective trial of 140 patients randomized to CyBorD with doxycycline showed no benefit for hematologic or cardiac progression-free survival, so the role of doxycycline has become less clear.^34^ We await results of an ongoing randomized control trial investigating the addition of doxycycline to bortezomib-based therapy.^35^

## Disease-Modifying Treatment of ATTR Cardiac Amyloidosis

Until recently, treatment of ATTR-CA was aimed at management of symptoms and disease-related complications. However, novel and experimental therapies for treatment of ATTR-CA wild-type (ATTRwt-CA) and hereditary/variant (ATTRv-CA) are emerging. Strategies for disease-modifying therapies target various steps along the ATTR-CA amyloid production process, including gene silencing to prevent hepatocyte TTR production, TTR stabilization to prevent TTR tetramer dissociation, anti-TTR antibodies, inhibition of TTR oligomer aggregation, and degradation of deposited ATTR fibrils with the goal to reverse the disease process, restore cardiac function, and consequently improve morbidity and mortality.

### ATTR Silencers

Two gene silencer therapies, patisiran and inotersen, are FDA approved for ATTRv polyneuropathy based on multicenter, international, randomized controlled phase 3 trials. Findings from cardiac subgroup analyses have prompted investigation for utility in ATTRv-CA and ATTRwt-CA.

Patisiran is a small interfering RNA that binds to the RNA-induced silencing complex and mediates cleavage of the transthyretin mRNA to prevent formation of TTR. It is administered intravenously every 3 weeks. The APOLLO randomized controlled trial evaluated the effect of patisiran for treatment of 225 patients with ATTRv polyneuropathy, 126 (56%) of whom had concomitant cardiac involvement.^36^ In addition to demonstrating slower progression of polyneuropathy at 18-months, a prespecified ATTRv-CA subgroup demonstrated improvement in NT-proBNP levels as well as echocardiographic parameters, including cardiac output, LV wall thickness, end-diastolic volume, and global longitudinal strain.^37^ The sub-study also demonstrated improved functional capacity (10-meter walk test) as well as decreased hospitalization and mortality with patisiran compared to placebo at 18-months. These results prompted the APOLLO-B phase 3 randomized controlled trial,^38^ which has completed enrollment with results pending, to evaluate patisiran for ATTRv-CA and ATTRwt-CA with the primary end point of 6-minute walk test (6MWT) performance and secondary end points of death and hospitalization at 12 months.

Inotersen is an antisense oligonucleotide inhibitor of transthyretin mRNA produced by the liver that is administered weekly by subcutaneous injection. The NEURO-TTR randomized controlled trial^39^ evaluated inotersen for 172 ATTRv polyneuropathy patients, 105 (61%) with cardiac involvement. Like APOLLO, NEURO-TTR demonstrated slower progression of polyneuropathy and improved quality of life; unlike APOLLO, there was no significant improvement in echocardiographic parameters within the prespecified cardiac subgroup. However, in an interim analysis of inotersen in a single-center open-label study,^40^ 33 patients with biopsy proven ATTR-CA without polyneuropathy (10 ATTRv-CA and 23 ATTRwt-CA) but with NYHA class I-III symptoms demonstrated a decrease in LV mass and improved exercise tolerance in 6MWT by 2 and 3 years of follow-up. A separate 24-month, single-center, open-label study of 50 patients with ATTRwt-CA or ATTRv-CA is ongoing.^41^

Although the subcutaneous use of inotersen may be preferable to the intravenously administered patisiran, post-hoc comparison of the APOLLO and NEURO-TTR trials demonstrates more improved neuropathy symptoms and a lower risk of adverse effects for patisiran compared with inotersen. NEURO-TTR demonstrated a higher frequency of serious adverse events with inotersen compared to placebo (32% vs 22%,), including thrombocytopenia and glomerulonephritis. In contrast, patisiran compared favorably with placebo in the APOLLO study. Patients in the patisiran group were premedicated with dexamethasone, acetaminophen, an H2-blocker, and an H1-blocker, which may have decreased adverse reactions.

From an historical perspective, it is important to note that a different RNA interference agent, revusiran, was evaluated for ATTRv-CA in the ENDEAVOUR phase 3 trial,^42^ which was prematurely terminated at 6 months due to increased mortality compared with placebo (14% vs 3%). Most deaths were determined to be due to heart failure, and a post-hoc safety investigation failed to reveal a causative mechanism.

Other ongoing randomized controlled trials include two novel silencer treatments for ATTR-CA (ATTRv-CA and ATTRwt-CA): vutrisiran and eplontersen. The HELIOS-B study^43^ is evaluating vutrisiran, another RNA interference therapy, with a primary composite end point of all-cause mortality and recurrent hospitalizations at 30 months. Like inotersen, vutrisiran is administered subcutaneously but with the advantage of dosing every 3 months rather than weekly. The CARDIO-TTRansform study^44^ is evaluating eplontersen, a ligand conjugated antisense oligonucleotide, for a primary end point of cardiovascular mortality and clinical events at 120 weeks. Eplontersen is administered every 4 weeks with an improved formulation targeting fewer side effects than inotersen.

Another exciting novel method for reduction of hepatocyte TTR production is the CRISPR-Cas9 system (clustered regularly interspaced short palindromic repeats and associated Cas9 endonuclease) gene editing treatment, which knocks out the TTR gene. In an open-label, multicenter study, intravenous injection of NTLA-2001 by the CRISPR-Cas9 method in six patients with ATTRv polyneuropathy was demonstrated to reduce serum concentrations of TTR by up to 87% by 4 weeks, with only mild adverse events in 3 patients.^45^ It is hoped that this one-time therapy provides durable knockdown of TTR with subsequent improvement in clinical outcomes. The initial pilot study in a small cohort suggests that treatment is safe and well tolerated with no signal for off-target gene editing. This is one of the first clinical applications for this gene editing technology, which appears particularly suited for ATTR because the protein is predominantly synthesized in the liver, and the CRISPR-Cas9 system can target the liver by encapsulation in a lipid nanoparticle with avidity for the low-density lipoprotein (LDL) receptor.

### ATTR Stabilizers

Diflunisal is a nonsteroidal anti-inflammatory (NSAID) medication that has demonstrated TTR stabilization in vitro^46^ but has only been studied in small, nonrandomized, mostly noncomparative single-arm prospective cohort trials of predominantly ATTRv-CA.^47,48,49,50^ Although these studies suggest stabilization of cardiac biomarkers and echocardiographic parameters, formal randomized controlled trials are needed to confirm these findings and determine an impact on morbidity and mortality. While diflunisal is generally considered safe and well tolerated, as an NSAID it is contraindicated in patients with significant thrombocytopenia and or renal dysfunction (glomerular filtration rate < 40 mL/min/1.73m^2^) and can cause gastrointestinal intolerance, bleeding, and heart failure exacerbation.

**Figure 2:**
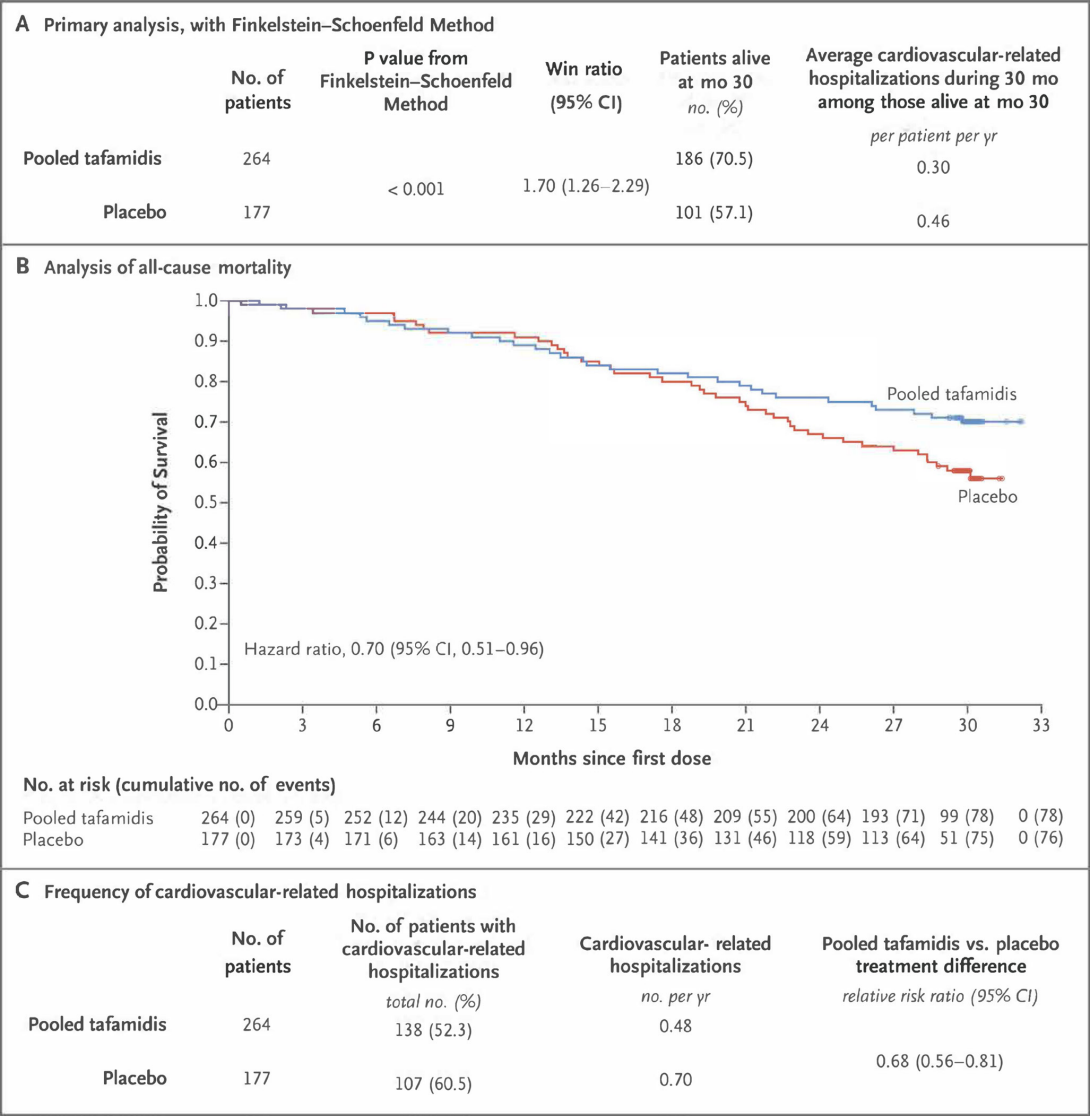
Primary and secondary outcomes of the ATTR-ACT (Safety and Efficacy of Tafamidis in Patients With Transthyretin Cardiomyopathy) Study investigation of pooled tafamidis (80 mg and 20 mg daily dosing) versus placebo. Panel A demonstrates the superiority of pooled tafamidis compared with placebo for the primary analysis using the Finkelstein-Schoenfeld method to hierarchically assess all-cause mortality and cardiovascular-related hospitalization. Panel B shows the Kaplan Meier survival curves demonstrating a reduction in all-cause mortality for pooled tafamidis compared to placebo, with curves diverting at 18-month follow-up (secondary outcome). Panel C shows the frequency of cardiovascular-related hospitalizations (secondary outcome). Reproduced with permission from the @Massachusetts Medical Society, Maurer et al.^51^

An analog of diflunisal, tafamadis has TTR stabilization characteristics but is not an NSAID. It is the only FDA-approved medication for both types of ATTR-CA since May 2019 after showing positive results in the ATTR-ACT study (Transthyretin Amyloidosis Cardiomyopathy Clinical Trial).^51^ This study evaluated 441 ATTR-CA patients (106 ATTRv-CA and 335 ATTRwt-CA) for treatment with oral tafamidis 80 mg vs tafamidis 20 mg vs placebo. In a pooled analysis, both doses of tafamidis were associated with a reduction in all-cause mortality (29.5% vs 42.9%, HR 0.70; CI 0.51-0.96) and cardiovascular hospitalizations (RR 0.68; CI 0.56-0.81) at 30 months (***Figure 2***). Secondary end points were notable for a lower rate of functional capacity decline by 6MWT distance and of quality-of-life decline by Kansas City Cardiomyopathy Questionnaire–Overall Summary score. There was a smaller increase in NT-proBNP levels and less worsening of circumferential and radial global strain without effect on other echocardiographic parameters, with no significant safety problems reported. However, there was a higher rate of cardiovascular-related hospitalizations in the patients with NYHA class III symptoms on treatment, which has been postulated to be due to longer survival in a more critical stage of disease. Nevertheless, poorer outcomes in more advanced disease states suggest the importance of early administration to prevent TTR fibril formation and concern for ineffectiveness for disease reversal with this strategy.

With FDA approval and a reassuring safety profile, tafamidis has become a mainstay of treatment for ATTR-CA. Although tafamidis has demonstrated significant efficacy in staving off disease progression, patients require life-long therapy, and the cost for many is prohibitive despite insurance, charity, and drug maker assistance programs. A cost-effectiveness model has indicated that the cost of tafamidis would need to be reduced by > 90% to be cost effective.^52^

Acoramidis, formerly known as AG-10, is a novel oral TTR stabilizer that mimics a protective TTR mutation (T119M). In ex vivo studies, acoramidis binds more strongly to TTR than tafamidis.^53^ Acoramidis is currently being investigated in the phase 3 randomized controlled trial, ATTRIBUTE-CM, for treatment of ATTR-CA NYHA Class I-III symptoms.^54^

#### Inhibition of Oligomer Aggregation and Disruption

Epigallocatechin-3-gallate has been shown to inhibit TTR amyloid fibril formation in vitro and in vivo in mice,^55,56^ although there have been only three small noncomparative prospective single-arm studies with follow-up for only 7 to 25 patients with ATTRwt-CA and ATTRv-CA.^57,58,59^ One 14-patient study demonstrated significant reduction in interventricular septal thickness in 12 patients, but it was not reproduced in the other studies.^59^ All cohorts demonstrated a very small reduction in LV mass by cardiac magnetic resonance imaging, and one study had a signal for mildly reduced T1 time without reduction in extracellular volume. Nevertheless, as with AL-CA, EGCG is often prescribed due to its benign safety profile.

### ATTR Degraders

While many of the other ATTR-CA treatments target interference of amyloid formation and deposition, an amyloid degradation strategy targets already deposited amyloid fibrils and promotes breakdown and clearance. Doxycycline and tauroursodeoxycholic acid (TUDCA) or ursodeoxycholic acid (UDCA) have been shown to remove amyloid deposits in preclinical studies.^60^ Only three noncomparative prospective single-arm studies have investigated this combination therapy, and they demonstrated modest results with a high attrition rate (~10%) due to esophageal and dermatologic intolerance from doxycycline.^61,62,63^ However, given the high cost of other disease-modifying therapies, doxycycline with TUDCA/UDCA is a desirable, cheaper option if it is determined to be effective in a phase 3 randomized controlled trial that has completed recruitment, with results pending.^64^

### ATTR Immunotherapy

Immunotherapy is also being investigated for treatment of ATTR-CA. PRX004 (Prothena) is an intravenously administered anti-TTR humanized monoclonal antibody. In preclinical studies, it was shown to promote clearance of insoluble amyloid fibrils through antibody-mediated phagocytosis and inhibition of amyloid fibril formation. A recent phase I open-label multicenter study was terminated early due to the COVID-19 pandemic; however, analysis of 7 patients with ATTRv-CA (NYHA Class I and II) demonstrated safety, improvement in neuropathic symptoms, and a modest improvement in global longitudinal strain (-1.21%) after 9 months of treatment.^65^ Another anti-TTR recombinant human monoclonal antibody, NI006, has also shown high affinity for binding to ATTRv and ATTRwt ex vivo and in vivo.^66^ It is currently under investigation for ATTR-CA in a phase 1, randomized, placebo-controlled double-blind trial^67^ with plans for a subsequent open-label extension to investigate safety and efficacy. From an historical perspective, it is important to note that the anti-serum amyloid P (SAP) component antibody, GSK2315698, initially garnered excitement for amyloid fibril clearance in a phase 1 trial (excluding those with cardiac involvement).^68^ However, due to an adverse risk/benefit profile in a phase 2 trial of ATTR-CA, its development was terminated.^69^

## General Cardiac Management

In addition to specific disease-modifying therapies, general management of CA focuses on treatment of heart failure symptoms and arrhythmias with the goal of optimizing quality of life. Since CA has traditionally been considered a rare and lethal condition, CA patients have been excluded from heart failure randomized controlled trials, so treatment recommendations have been limited to expert consensus and small cohort studies.

The cornerstone of treatment for CA is loop diuretic therapy. As CA patients have a very narrow window of volume optimization due to small, stiff ventricles with limited stroke volume, they can easily become volume overloaded with resultant pulmonary, hepatic, and renal congestion. Conversely, with slight overdiuresis, an already decreased stroke volume is further reduced, resulting in kidney, brain, and muscle hypoperfusion contributing to renal insufficiency and profound fatigue. Due to frequent gut edema, the more bioavailable loop diuretics, bumetanide and torsemide, are commonly used over furosemide and are sometimes paired with a mineralocorticoid receptor antagonist (MRA) with close monitoring of potassium and renal function. Compression stockings are also used to mobilize edema and to assist with frequent co-occurrence of autonomic dysfunction, particularly with AL-CA and ATTRv-CA. Patients are instructed to limit sodium intake and to closely monitor daily weights and blood pressure since subtle volume changes can have large hemodynamic effects.

There is no established guideline-directed medical therapy for heart failure with preserved ejection fraction or reduced ejection fraction in patients with CA. Angiotensin receptor-neprilysin inhibitors, angiotensin receptor blockers, angiotensin-converting enzyme inhibitors, MRAs, and sodium-glucose cotransporter-2 inhibitors have not been studied in this population but may be poorly tolerated or even contraindicated due to autonomic dysfunction, oscillating fluid balance, and renal dysfunction. Vasoconstrictors such as midodrine may be needed to manage hypotension, particularly in patients with AL-CA and ATTRv-CA, where autonomic dysfunction can be debilitating.^70,71^ Since orthostatic hypotension can be challenging to treat, droxidopa may be used as an adjunct to midodrine and compression stockings and was recently approved specifically for management of symptomatic neurogenic orthostatic hypotension.^72^ Pyridostigmine, the acetylcholinesterase inhibitor, can also be used but has not been specifically studied in CA.^73^ Fludrocortisone should be avoided due to risk of volume retention.

Since CA patients have small stroke volumes, they are dependent on increases in heart rate to augment cardiac output. However, because of extensive fibrosis, CA patients are particularly prone to conduction system disease, syncope, and death. Thus, treatment with atrioventricular nodal blocking agents must be used cautiously since they can contribute to bradyarrhythmias and worsening low-output heart failure. Nondihydropyridine calcium channel blockers are typically contraindicated in CA patients because they bind avidly to amyloid fibrils, have profound negative inotropic effects, and exacerbate bradyarrhythmias.^74^

With development of high-degree heart block with or without symptoms, pacemaker implantation is a Class 1-B recommendation.^75^ Since high pacing burden has been associated with deleterious remodeling and progressive heart failure symptoms in ATTR-CA, biventricular pacemaker upgrade may be necessary and has been shown to provide functional improvement.^76^ The role of implantable cardioverter defibrillators (ICD) is less clear and has been predominantly studied in AL-CA. While appropriate ICD therapies for ventricular arrhythmias are common, particularly in those with less-advanced CA, ICD implantation has not been shown to improve survival.^77^ Nevertheless, if a patient with CA has a sudden cardiac arrest with a reasonable survival of greater than 1 year, ICD implantation carries a class I-C recommendation.^75^

Due to atrial enlargement from diastolic dysfunction and atrial fibrosis, atrial fibrillation is common and occurs in up to 44% of patients.^78^ While there is no clear evidence to support a rate or rhythm control strategy in CA, rhythm control with amiodarone, and less commonly dofetilide or sotalol, is a class IIb-C recommendation due to frequent beta blocker intolerance. Digoxin sensitivity and toxicity was reported over 40 years ago and attributed to potentiated effects from myocardial binding^79^ and decreased renal clearance from concomitant renal dysfunction. However, a recent retrospective study of digoxin use in AL-CA suggests possible utility at lower doses and with close serologic monitoring.^80^ Cardioversion and ablation can also be considered in select cases and may be most beneficial in earlier disease states, although there is limited data in this population (Class IIb-C recommendation). Since left atrial appendage thrombosis occurs in up to one-third of patients, even in sinus rhythm without a diagnosis of atrial fibrillation, anticoagulation is prescribed for a diagnosis of atrial fibrillation regardless of the CHA2DS2-VASc score and should even be considered in select patients without atrial arrhythmias.^81,82^ Either warfarin or a direct oral anticoagulant may be safely prescribed. The benefit of atrial appendage occluder device placement in this population has also not been evaluated but can be considered for appropriate patients.

ATTRwt-CA has been reported in up to 16% of patients undergoing transcatheter aortic valve replacement (TAVR).^83^ Two recent studies demonstrate safety and similar outcomes for TAVR for ATTRwt-CA with aortic stenosis compared to those with lone aortic stenosis.^84,85^

## Advanced Heart Failure Therapies

Due to the characteristically small and hypertrophied LV with frequent right ventricular involvement, LV assist devices (LVAD) are often not feasible for CA and have been associated with poor outcomes in small studies.^86^ Total artificial heart overcomes these barriers to LVAD and was shown to serve as a successful bridge to cardiac transplantation in a small retrospective study.^87^

Increasingly, select patients with advanced cardiac disease, with or without renal disease, are considered for heart or combined heart-kidney transplantation, with improving survival rates.^88,89,90,91^ Cardiac transplantation provides clinical improvement to facilitate subsequent chemotherapy and autologous stem cell transplantation for those with AL-CA.^92^ For patients with ATTRv-CA with neuropathy, heart-liver transplant has historically been performed to prevent progression of debilitating neuropathy but is now superseded by the availability of gene-silencing therapies.

## Conclusion

While the prognosis of CA is still poor relative to other forms of cardiomyopathies, the stars have started to align for improvement in CA prognosis with significant advances in diagnostic methods as well as novel and specific disease-modifying therapies. We eagerly await the results of ongoing randomized controlled trials as well as inclusion of this population in future studies to enhance our approach to this disease.

## Key Points

- While autologous stem cell transplantation was previously favored for up-front treatment of patients with light chain amyloidosis (AL) with acceptable risk, daratumumab in combination with a bortezomib-based chemotherapy regimen recently became the first treatment approved by the US Food and Drug Administration (FDA) for AL amyloidosis as it has demonstrated excellent hematologic and organ response in newly diagnosed AL.
- There is hope that daratumumab monotherapy may serve as a safe, tolerable, and efficacious treatment for those with advanced AL cardiac amyloidosis and is currently under investigation.
- The transthyretin (TTR) tetramer stabilizer, tafamidis, is the only FDA-approved treatment for transthyretin cardiac amyloidosis (ATTR-CA). However, there are promising novel therapies on the horizon that target pathogenic TTR fibril formation including TTR gene silencers, TTR tetramer stabilizers, oligomer aggregation inhibitors, anti-TTR fiber antibodies, and TTR amyloid degraders (ongoing clinical trials).
- For end-stage CA, advanced interventions including heart or heart-kidney transplantation may need to be considered. For patients with hereditary ATTR-CA with neuropathy, heart-liver transplant has been historically performed to prevent progression of debilitating neuropathy but is now superseded by the availability of gene-silencing therapies such as patisiran or inotersen.
- Despite the evolution of treatment options, CA management remains complex due to patient frailty and therapeutic side effects in advanced cardiac disease states.
